# Supplementary material for: Association between oral microbiome and seven types of cancers in East Asian population: a two-sample Mendelian randomization analysis
Source: Front Mol Biosci. 2023 Nov 21;10:1327893. doi: 10.3389/fmolb.2023.1327893 (PMC10702768; doi:10.3389/fmolb.2023.1327893)
Supplement: Supplementary file 1 [file DataSheet1.ZIP › Supplementary Materials/MR plots of saliva/saliva═╝/Prostate cancer/pheno.1102_to_prostate cancer_forest.pdf]

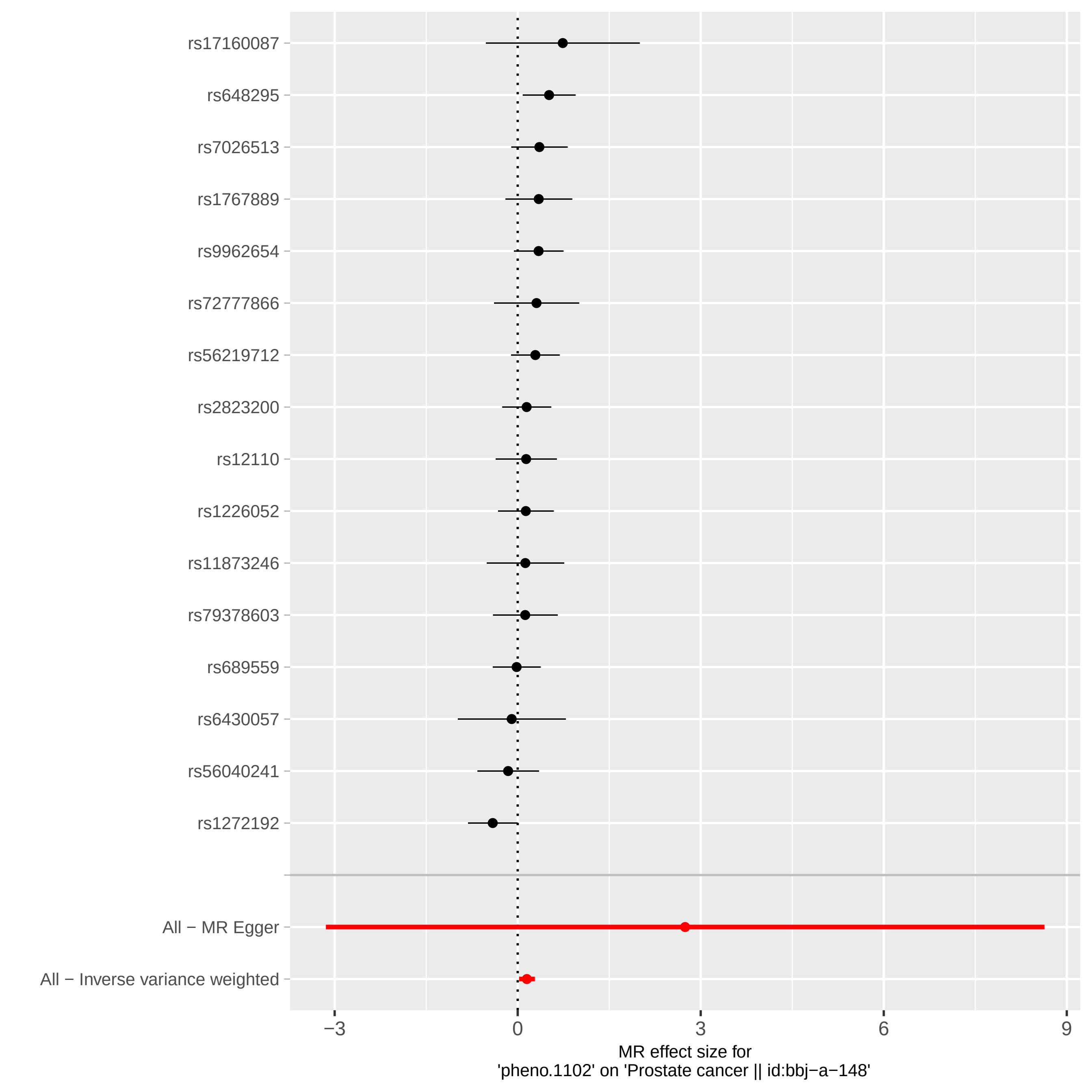
rs17160087
rs648295
rs7026513
rs1767889
rs9962654
rs72777866
rs56219712
rs2823200
rs12110
rs1226052
rs11873246
rs79378603
rs689559
rs6430057
rs56040241
rs1272192
All − MR Egger
All − Inverse variance weighted
−3
0
3
6
9
MR effect size for
'pheno.1102' on 'Prostate cancer || id:bbj−a−148'
